# FedGraphHE: A privacy-preserving federated graph neural network framework with dynamic homomorphic encryption and robust aggregation

**DOI:** 10.1371/journal.pone.0339881

**Published:** 2026-01-05

**Authors:** Aocheng Zuo, Zhanshen Feng, Yuan Ping, Shaohua Tao, Haonan Sun, Yange Chen

**Affiliations:** 1 School of Information and Control Engineering, Jilin University of Chemical Technology, Jilin, China; 2 School of Information Engineering, Xuchang University, Xuchang, China; 3 Henan Province Engineering Technology Research Center of Big Data Security and Application, Xuchang, China; 4 Henan International Joint Laboratory of Polarization Sensing and Intelligent Signal Processing, Xuchang, China; University of Education, PAKISTAN

## Abstract

Federated learning (FL) enables collaborative model training across distributed intelligent devices while preserving data privacy. In smart healthcare networks, medical institutions can jointly learn from distributed patient data using graph neural networks (GNNs). This approach improves diagnostic accuracy without compromising patient confidentiality. However, federated GNNs face substantial challenges. These include gradient privacy vulnerabilities, computational overhead from homomorphic encryption, and susceptibility to Byzantine attacks. This paper presents FedGraphHE, a privacy-preserving federated GNN framework for secure collaborative intelligence. Our methodology integrates three synergistic modules. First, Dynamic Adaptive Partitioned Homomorphic Encryption (DAPHE) optimizes gradient transmission. Second, Hierarchical Multi-scale Adaptive Graph Transformer (HMAGT) enables encryption-aware graph processing. Third, Federated Robust Aggregation via Homomorphic Inner Product (FRAHIP) provides Byzantine-resilient aggregation. Experimental results demonstrate FedGraphHE’s effectiveness across multiple scenarios. The framework consistently outperforms existing privacy-preserving methods on citation network benchmarks (Cora, CiteSeer, PubMed). It achieves 98.18% classification accuracy on medical imaging datasets (ISIC 2020), and reduces communication costs by approximately 25% compared to existing homomorphic encryption baselines. The framework maintains over 95% accuracy under Byzantine attacks, establishing it as an effective solution for privacy-sensitive collaborative learning applications.

## 1 Introduction

Graph-structured data have become fundamental to modern medical artificial intelligence research, with GNNs demonstrating exceptional performance in pathological image analysis [1], multi-center electronic health records [2], and medication recommendation systems [3]. However, the sensitive nature of medical data and stringent privacy regulations [4] (e.g. the General Data Protection Regulation [GDPR] and the Health Insurance Portability and Accountability Act [HIPAA]) create significant barriers to cross-institutional collaboration. Traditional centralized approaches face inherent limitations, including data silos and high communication costs, making it challenging to simultaneously achieve effective collaboration and privacy preservation [5].

Federated learning provides a promising solution for privacy-preserving training on distributed graph data [6]. However, directly applying federated learning to GNNs presents significant technical challenges. The message passing mechanisms in GNNs cause each node to encode information from its neighbors, making gradient updates particularly vulnerable to privacy attacks that may expose both node features and graph topology [7]. To address these privacy concerns, the Cheon-Kim-Kim-Song (CKKS) homomorphic encryption scheme [8] offers strong privacy guarantees for real-valued computations. However, it introduces substantial computational and communication overhead for high-dimensional graph parameters. Furthermore, existing homomorphic encryption approaches struggle with the complex nonlinear operations inherent in GNNs, often requiring computationally expensive polynomial approximations that compromise both accuracy and efficiency [9]. Moreover, the heterogeneous and non-independent and identically distributed (non-IID) characteristics inherent in graph data render models more vulnerable to poisoning attacks [10], while traditional Byzantine robustness aggregation methods cannot function under encryption constraints as they require plaintext gradient access for similarity computation and outlier detection.

Graph-structured data in healthcare, finance, and collaborative research exhibits three compounding challenges absent in conventional federated learning: recursive neighbor aggregation causes gradients to encode both node features and graph topology, creating privacy risks beyond individual data points; irregular connectivity patterns and power-law degree distributions render homomorphic encryption substantially more expensive than on regular architectures; and non-IID graph characteristics amplify vulnerability to Byzantine attacks through adversarial embedding propagation. Recent advances have addressed individual aspects of these challenges. For instance, SecureGraphFL [11] integrated privacy with robustness for spatiotemporal graphs; cryptographic methods [12,13] provided strong guarantees but incurred substantial computational overhead; and federated frameworks [14–16] improved efficiency but lacked encrypted-domain robustness. However, no existing approach jointly optimizes privacy, efficiency, and robustness for heterogeneous graphs with irregular structures. To address these limitations, this paper introduces **FedGraphHE**, a privacy-preserving federated graph neural network framework. The primary contributions of this work are as follows:

- **Dynamic Ring Dimension Optimization.** The framework introduces a federation-wide parameter optimization mechanism that dynamically adjusts CKKS encryption parameters based on gradient dimensions across participants. By maximizing slot utilization efficiency and implementing adaptive partitioning strategies, this mechanism reduces communication costs by approximately 25% in federated environments with heterogeneous gradient dimensions.
- **Encryption-Aware Graph Architecture.** The framework employs a multi-scale graph processing architecture that captures multi-hop neighborhood information through parallel single-step aggregation while constraining homomorphic multiplication depth. This encryption-aware design enables practical privacy-preserving training while demonstrating superior performance compared to existing privacy-preserving methods.
- **Encrypted Domain Robust Aggregation.** A scalar-based Byzantine detection mechanism utilizing homomorphic inner product computation identifies malicious participants while preserving gradient privacy. The reputation-weighted secure aggregation maintains robust model performance under Byzantine attacks, achieving accuracy exceeding 95% under f < *K*/3 malicious participants.

The remainder of this paper is organized as follows. Sect 2 reviews related work. Sect 3 details the design of the **FedGraphHE** framework, including the system model and three core modules. Sect 4 presents an experimental evaluation that validates the performance and security properties. Sect 5 concludes with a summary of contributions and future research directions. Key notations used throughout this paper are defined in Table 1.

**Table 1:** List of symbols.

Notation	Description
*K*	Total number of clients
𝒞	Set of clients
*D*_*i*_	The local dataset of client *C*_*i*_
*w*_*i*_	Aggregation weight for client *i*
$\omega^{\left(t\right)}$	The global model parameters at round *t*
$\omega_{i}^{\left(t\right)}$	The local model parameters of client *i* at round *t*
${ℒ}_{i}(\omega )$	The loss function of client *C*_*i*_
*N*	CKKS polynomial ring dimension
Δ	CKKS scaling factor
*Q*	Coefficient modulus chain
${𝐠}_{i}^{\left(t\right)}$	Gradient of client *i* at round *t*
$\left[{𝐠}_{i}^{\left(t\right)}\right]$	Encrypted gradient of client *i* at round *t*
${\bar{𝐠}}^{\left(t\right)}$	Consensus gradient at round *t*
$\left[{\bar{𝐠}}^{\left(t\right)}\right]$	Encrypted consensus gradient
*[s*_*i*_ *]*	Encrypted inner product scalar
*s*_*i*_	Decrypted inner product scalar
*[s*_0_ *]*	Encrypted norm of consensus gradient
*s*_0_	Decrypted norm of consensus gradient
*d*_*i*_	Gradient dimension of client *i*
𝒟	Set of client gradient dimensions $\left\{d_{1},d_{2},\ldots ,d_{K}\right\}$
*FS*_*i*_	Consistency score for client *i*
$rep_{i}^{\left(t\right)}$	Reputation value of client *i* at round *t*
*f*	Maximum number of malicious clients
ℋ	Set of honest clients
ℳ	Set of malicious clients
θ	Consistency threshold
δ	Threshold tolerance parameter
γ	Reputation adjustment step size
β	Temperature parameter for softmax
η	Learning rate

## 2 Related works

### Privacy-preserving federated GNNs

Graph Neural Networks (GNNs) demonstrate superior performance through recursive neighbor aggregation [7]. However, this mechanism simultaneously increases privacy exposure because shared gradients may encode both node attributes and structural information. Existing studies in federated graph learning have explored different aspects of this challenge, yet comprehensive solutions that jointly consider privacy, robustness, and computational efficiency remain limited.

Early privacy-preserving GNN approaches primarily focused on encrypted inference in centralized or single-party settings. Ran et al. [13] introduced CryptoGCN with an Adjacency Matrix Aware (AMA) representation to support homomorphic GNN inference, while Wang et al. [12] proposed SecGNN to enable encrypted training. While these methods provide strong privacy guarantees, they incur substantial computational overhead, rendering them impractical for the resource-constrained clients typical in large-scale federated deployments.

Subsequent federated graph learning frameworks have made significant strides in distributed optimization. Chen et al. [14] proposed FedGraph with efficient sampling strategies, while Wu et al. [15] introduced FedPerGNN for privacy-preserving personalization through graph expansion, and Liu et al. [16] (ESA-FedGNN) utilized FFT-based secret sharing to optimize aggregation, achieving O(logNlog(logN)) complexity. Zhang et al. [17] extended these efforts to non-IID graph data. However, these approaches typically rely on the honest-but-curious threat model, failing to address active Byzantine attacks.

To address the need for attack resilience, Chen et al. [11] recently proposed SecureGraphFL, a framework utilizing an actor-critic network for client selection and attention-based aggregation. While SecureGraphFL significantly outperforms standard federated approaches in resilience, it is primarily tailored for spatiotemporal graphs, such as traffic prediction networks, characterized by inherent structural regularity. Its applicability to general, heterogeneous graph structures with irregular connectivity remains a challenge, particularly when balancing high-grade privacy with model utility.

The necessity for a solution capable of handling such structural irregularities is further underscored by recent advances in graph neural architecture search (NAS). Wang et al. [18] proposed ABG-NAS, revealing that real-world graph datasets exhibit extreme diversity in node degree distributions and sparsity-density variations. These findings highlight a critical motivation for our work: standard privacy-preserving techniques designed for grid-like image data perform poorly on graphs. The structural heterogeneity identified by Wang et al. drastically complicates homomorphic encryption, as encrypted operations on irregular topologies are computationally demanding.

Consequently, existing approaches have not fully addressed the challenge of jointly optimizing privacy, efficiency, and robustness for heterogeneous graphs with irregular structures. While methods exist for specific tasks such as medical diagnosis [19] or traffic prediction [11], they rarely provide comprehensive solutions that simultaneously protect node features, secure graph topology, maintain computational efficiency, and defend against Byzantine threats.

### Homomorphic encryption optimizations

Building upon the federated GNNs privacy challenges identified above, homomorphic encryption emerges as a promising solution, though it introduces new optimization challenges. The CKKS homomorphic encryption scheme exhibits fundamental limitations regarding noise accumulation and ciphertext packing capacity [20]. While recent advances have substantially improved practical performance, graph-specific optimizations remain underexplored.

Significant progress has been achieved in bootstrapping optimization. Jung et al. [21] demonstrated substantial performance improvements in CKKS bootstrapping. However, recent research by Al Badawi and Polyakov [22] revealed that bootstrapping operations remain the most computationally intensive component in FHE schemes, with CKKS bootstrapping achieving higher throughput than other schemes but still incurs significant latency overhead for applications requiring frequent noise management.

Parameter management and slot utilization have experienced notable advances. Recent work by Cheon et al. [23] introduced Grafting, a novel approach that decoupled scale factors from the modulus in RNS-CKKS. This approach addressed the rigid coupling that previously imposed design constraints and reduced precision flexibility. Pan et al. [24] developed segmented adaptive CKKS encryption specifically for federated learning scenarios. For GNNs, Ran et al. [25] proposed parallel-packed homomorphic encryption optimized for efficient graph convolutional network inference. Additionally, Zhang et al. [26] addressed communication efficiency through BatchCrypt, which batched quantized gradients for single-pass homomorphic encryption. However, the gradient quantization introduces approximation errors that may prove problematic for graph data’s irregular sparsity patterns. Despite these advances, existing homomorphic encryption optimizations were developed independently of federated graph learning requirements. Graph data sparsity, power-law degree distributions, and irregular computation patterns require specialized optimization strategies that current methods fail to adequately address.

### Robust aggregation under encryption

The vulnerability of federated learning to Byzantine attacks has motivated extensive research in robust aggregation methods. However, the intersection with privacy preservation, particularly under homomorphic encryption, presents fundamental challenges that extend beyond traditional Byzantine fault tolerance.

Traditional robust aggregation methods operate on plaintext gradients. Blanchard et al. [27] performed gradient selection using nearest neighbor distance analysis, providing Byzantine tolerance through similarity-based selection. Pillutla et al. [28] proposed robust geometric median-based aggregation with theoretical convergence guarantees. However, the inner product manipulation attack proposed by Xie et al. [29] demonstrated a fundamental weakness in robust aggregation based on similarity. These traditional defenses face increasingly sophisticated attack strategies, including enhanced model poisoning attacks [30], data poisoning attacks [31], and gradient inversion attacks [32], which exploit various vulnerabilities in federated learning systems.

Robust aggregation under encryption constraints represents a critical research gap. Recent methods like ELSA [33] provide secure aggregation against malicious actors, but its reliance on secret sharing and distributed trust models constrains deployment flexibility and limits compatibility with homomorphic encryption frameworks. While the RFLPA framework [34] advanced secure aggregation through efficient secret sharing techniques, achieving significant performance improvements over prior work, the fundamental challenge of enabling robust aggregation directly within homomorphic encryption schemes remains unresolved. In the context of GNNs, these challenges are amplified by gradient interdependence, wherein malicious participants can inject adversarial node embeddings that propagate through message passing layers. Existing approaches typically address robustness or privacy individually, creating a need for methods capable of simultaneously handling both requirements under homomorphic encryption constraints.

## 3 Methodology

### Ethics statement

**Fig 1:**
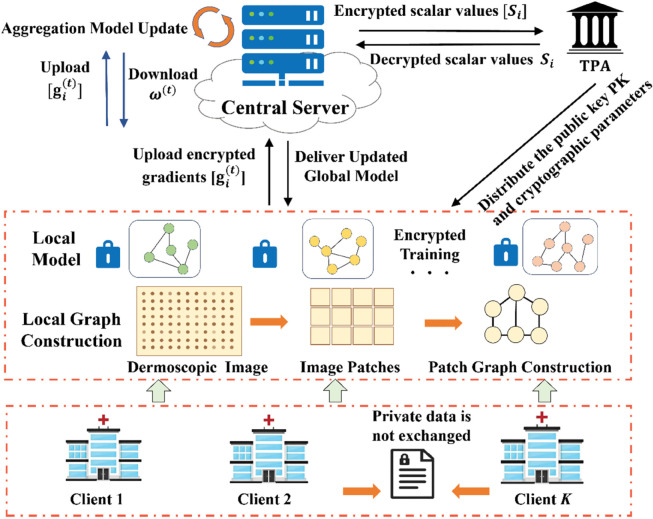
Medical collaboration graph.

**Fig 2:**
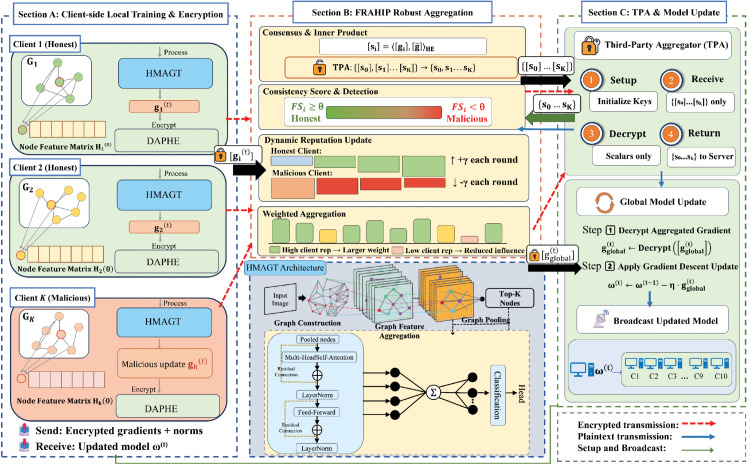
Overview of the FedGraphHE framework architecture.

This computational study does not require ethics committee approval as it involves purely algorithmic research without human participants. No informed consent was obtained as no human subjects were involved. All datasets used (ISIC 2020, Cora, CiteSeer, PubMed) are publicly available and were fully anonymized prior to public release. Figure Clarification: All figures in this manuscript (including Figs 1 and 2) use schematic representations or stock illustrations to depict the system architecture and medical scenarios. No real patient images, clinical photographs, or personally identifiable information are included.

### Problem formulation

#### System architecture.

As illustrated in Fig 1, the proposed framework constructs a federated learning system consisting of a central server (CS), a set of *K* client nodes $𝒞=\{C_{1},C_{2},\ldots ,C_{K}\}$, and a third party (TPA). The system operates in multiple training rounds, where the CS broadcasts the current global model, clients perform local computation, and encrypted updates are transmitted back to the server. Through distributed optimization, the framework enables collaborative model training while ensuring that each client’s private data remain strictly local and confidential, thereby collaboratively learning a global model without exposing raw information.

The system entities are defined as follows:

- **Clients:** Each client *C*_*i*_ maintains a private local dataset *D*_*i*_, where i∈{1,…,K}. Their primary responsibility is to receive the global model, perform local training computations and model updates, and transmit encrypted updates to the central server.
- **CS:** Operating under the *honest but curious* adversarial model, the CS coordinates the federated training process by distributing the global model and aggregating encrypted model updates from participating clients. The CS does not have access to raw data or plaintext model parameters.
- **TPA:** The TPA serves as an independent entity responsible for cryptographic key management. It remains uninvolved in the model training process. Its exclusive role is to manage cryptographic keys for the encryption scheme and execute required decryption operations.

#### Threat model and security requirements.

FedGraphHE enables collaborative GNN training on distributed graph data while preserving privacy by avoiding raw data sharing. The following assumptions apply to each entity:

- **Clients:** At most *f*<*K*/3 clients are malicious and may submit arbitrary gradients for model poisoning or collusion attacks. Honest clients follow the protocol faithfully and do not collude with the central server.
- **Semi-honest Central Server:** The CS follows the protocol but attempts to infer private information from encrypted gradients and computation patterns. While unable to decrypt ciphertexts, it may analyze encrypted data distributions.
- **Semi-honest TPA:** The TPA correctly executes decryption operations but may attempt to infer information from decrypted scalar values. Information exposure is limited by restricting TPA access to only 𝒪(K) encrypted scalars per round, with no access to high-dimensional gradients. The TPA does not collude with the CS or malicious clients.

The framework maintains three security requirements: (1) concealing each client’s local graph data and gradients from the server and other clients; (2) ensuring robust aggregation against malicious clients while preserving gradient privacy; and (3) minimizing information leakage through homomorphic computations, including limiting TPA information access. The threat model assumes secure communication channels and considers both privacy attacks targeting gradient information and Byzantine attacks compromising model integrity.

### FedGraphHE framework overview

FedGraphHE integrates federated learning with GNNs for collaborative modeling in privacy-sensitive domains. The framework employs three synergistic modules: DAPHE for privacy-preserving gradient transmission, HMAGT for efficient graph representation learning, and FRAHIP for secure aggregation under malicious scenarios. Their interplay forms a unified processing flow: DAPHE establishes a common encrypted computational domain through federation-wide ring-dimension alignment; HMAGT performs multi-scale feature aggregation and produces structured ciphertext gradients with constrained homomorphic depth; FRAHIP operates directly on these ciphertexts to conduct consistency evaluation and Byzantine-resilient aggregation without exposing gradient information. Critically, graph topology remains strictly local—only encrypted node feature representations and gradients are transmitted, ensuring that adjacency matrices and structural connectivity patterns are never exposed. This design ensures that encrypted gradients from heterogeneous clients remain compatible, analyzable, and securely aggregatable throughout the federated pipeline.

FedGraphHE operates through a four-stage protocol per communication round:

- **Model Distribution.** CS broadcasts global model parameters to all clients.
- **Local Training & Encryption.** Each client trains locally using HMAGT, computes gradients, and encrypts them via DAPHE.
- **Secure Aggregation.** CS executes FRAHIP to compute consistency scores, update reputations, and perform weighted aggregation in the encrypted domain.
- **Global Update.** CS updates the global model using aggregated gradients.

The complete algorithmic specification is presented in Algorithm 1, while the overall system architecture is depicted in Fig 2. Technical specifications for each module are detailed in the following sections.

**Algorithm 1 FedGraphHE framework execution workflow.**

**Require:** Client set $𝒞=\{C_{1},\ldots ,C_{K}\}$, rounds *T*

**Ensure:** Global model $\omega^{\left(T\right)}$

1: **Initialize:** $\omega^{\left(0\right)}$, client reputations $\{rep_{i}^{\left(0\right)}=1.0{\}}_{i=1}^{K}$

2: **for** *t* = 1 to *T* **do**

3:   **Step 1:** CS broadcasts $\omega^{\left(t-1\right)}$ to all clients

4:   **Step 2:** Each client *C*_*i*_ in parallel executes:

5:    Initialize local model: $\omega_{i}^{\left(t\right)}\leftarrow \omega^{\left(t-1\right)}$

6:    Local training using HMAGT architecture:

$\omega_{i}^{\left(t\right)}\leftarrow$ LocalUpdate($\omega_{i}^{\left(t\right)},D_{i}$)

7:    Compute gradients: ${𝐠}_{i}^{\left(t\right)}\leftarrow \omega_{i}^{\left(t\right)}-\omega^{\left(t-1\right)}$

8:    DAPHE encryption: $[{𝐠}_{i}^{\left(t\right)}]\leftarrow$ DAPHE.Encrypt(${𝐠}_{i}^{\left(t\right)}$)

9:    Send encrypted gradients $\left[{𝐠}_{i}^{\left(t\right)}\right]$ and $\|{𝐠}_{i}^{\left(t\right)}{\|}^{2}$ to CS

10:   **Step 3:** FRAHIP robust aggregation protocol

11:    Compute consensus: $\left[{\overset{―}{𝐠}}^{\left(t\right)}]\leftarrow \frac{1}{K}\sum_{i=1}^{K}[{𝐠}_{i}^{\left(t\right)}\right]$

12:    Compute homomorphic inner products: $[s_{i}]\leftarrow \langle [{𝐠}_{i}^{\left(t\right)}],[{\overset{―}{𝐠}}^{\left(t\right)}]\rangle_{HE}$

13:    Send $\left\{[s_{0}],[s_{1}],\ldots ,[s_{K}]\right\}$ to TPA for decryption

14:    TPA returns decrypted scalars $\left\{s_{0},s_{1},\ldots ,s_{K}\right\}$ to CS

15:    Compute consistency scores: $FS_{i}\leftarrow \frac{s_{i}}{\sqrt{\|{𝐠}_{i}^{\left(t\right)}{\|}^{2}\cdot s_{0}}}$

16:   **Update Reputations:**

17:   **for** *i* = 1 to *K* **do**

18:    **if** $FS_{i}\geq \theta$ **then**

19:     $rep_{i}^{\left(t\right)}\leftarrow \min(rep_{i}^{\left(t-1\right)}+\gamma ,rep_{\max})$

20:    **else if** $FS_{i}<\theta -\delta$ **then**

21:     $rep_{i}^{\left(t\right)}\leftarrow \max(rep_{i}^{\left(t-1\right)}-\gamma ,rep_{\min})$

22:    **else**

23:     $rep_{i}^{\left(t\right)}\leftarrow rep_{i}^{\left(t-1\right)}$

24:    **end if**

25:   **end for**

26:   **Step 4:** Compute aggregation weights

27:    $w_{i}\leftarrow \frac{\exp(\beta \cdot rep_{i}^{\left(t\right)})}{\sum_{j=1}^{K}\exp(\beta \cdot rep_{j}^{\left(t\right)})}$

28:   **Step 5:** Weighted aggregation in encrypted domain

29:    $\left[{𝐠}_{global}^{\left(t\right)}]\leftarrow \sum_{i=1}^{K}w_{i}\cdot [{𝐠}_{i}^{\left(t\right)}\right]$

30:    TPA decrypts: ${𝐠}_{global}^{\left(t\right)}\leftarrow$ Decrypt($\left[{𝐠}_{global}^{\left(t\right)}\right]$)

31:   **Step 6:** Update global model

32:    $\omega^{\left(t\right)}\leftarrow \omega^{\left(t-1\right)}-\eta \cdot {𝐠}_{global}^{\left(t\right)}$

33: **end for**

34: **return** $\omega^{\left(T\right)}$

### Dynamic adaptive partitioned homomorphic encryption

In federated graph learning, the dimensionality of local gradients differs substantially across clients due to heterogeneous model structures and graph sizes. Existing encrypted federated learning systems typically rely on static CKKS parameterization, where a fixed ring dimension is applied uniformly to all participants. Although straightforward, this configuration forces all clients to operate under the worst-case parameter setting dictated by the largest gradient dimension, leading to severe slot under-utilization for smaller models. When gradients exceed the available ciphertext capacity, static schemes further suffer from excessive padding overhead arising from naive sequential partitioning.

The key insight underlying DAPHE is to maximize slot utilization while minimizing communication overhead. For client *i* with gradient dimension *d*_*i*_, slot utilization efficiency under ring dimension *N* is quantified as:

$$\eta_{i}(N)=\frac{d_{i}}{\lfloor N/2\rfloor },\;\text{subject to }\eta_{i}(N)\leq 1$$ (1)

The optimization objective maximizes the average slot utilization $\bar{\eta }=\frac{1}{K}\sum_{i=1}^{K}\min(\eta_{i}(N),1)$ while controlling the communication overhead induced by excessive partitioning.

To achieve this, DAPHE employs a dynamic ring-dimension selection mechanism that balances capacity requirements with utilization efficiency, as shown in Eq (2). The threshold values (0.7 and 0.6) represent empirically optimized balance points between utilization efficiency and computational costs:

$$N^{*}(𝒟)=\left\{ \begin{array}{cc} 8192 & \text{if }\max(𝒟)\leq 2867\text{ and }\bar{\eta }(8192)\geq 0.7,\\ 16384 & \text{if }\max(𝒟)\leq 5734\text{ and }\bar{\eta }(16384)\geq 0.6,\\ 32768 & \text{otherwise}. \end{array}\right.$$ (2)

Once the optimal ring dimension N^*^ is selected, all ciphertexts reside in the same polynomial ring ${\mathbb{Z}}_{q}[X]/(X^{N^{*}}\;+\;1)$, ensuring compatibility for homomorphic aggregation without requiring ring conversion. The available slot capacity per ciphertext is $\text{slots}=\lfloor N^{*}/2\rfloor$.

When a client’s gradient dimension exceeds this capacity ($d_{i}>\text{slots}$), naive fixed-size partitioning results in significant waste in the final block. To mitigate this, DAPHE adopts an adaptive block-size strategy that evenly distributes gradient elements across ciphertexts:

$${\text{BlockSize}}_{i}=\left\{ \begin{array}{cc} \text{slots} & \text{if }d_{i}\leq 1.2\times \text{slots},\\ \left\lfloor \frac{d_{i}}{\left\lceil d_{i}/\text{slots}\right\rceil }\right\rfloor & \text{otherwise}. \end{array}\right.$$ (3)

This adaptive design substantially reduces padding overhead while maintaining full compatibility with the unified ring-dimension configuration. The complete procedure is summarized in Algorithm 2, which includes federation- wide parameter selection followed by client-side adaptive encryption.

**Algorithm 2 DAPHE: Dynamic adaptive partitioned encryption.**

**Require:** Gradient dimensions $𝒟=\{d_{1},\ldots ,d_{K}\}$

1: Compute optimal $N^{*}(𝒟)$ using Eq (2)

2: Broadcast $\left(N^{*},\;\text{slots}=\lfloor N^{*}/2\rfloor \right)$ to all clients

3: **for** each client *i* in parallel **do**

4:   Compute ${\text{BlockSize}}_{i}$ using Eq (3)

5:   **if** $d_{i}\leq \text{slots}$ **then**

6:    ${𝒞}_{i}\leftarrow \{\text{Encrypt}(g_{i})\}$

7:   **else**

8:    Partition *g*_*i*_ into $m_{i}=\lceil d_{i}/{\text{BlockSize}}_{i}\rceil$ blocks

9:    **for** *j* = 1 to *m*_*i*_ **do**

10:     ${𝒞}_{i}\leftarrow {𝒞}_{i}\cup \{\text{Encrypt}({\text{block}}_{i,j})\}$

11:    **end for**

12:   **end if**

13:   Send ${𝒞}_{i}$ to server

14: **end for**

15: **return** $\{{𝒞}_{i}{\}}_{i=1}^{K}$

The novelty of DAPHE lies in combining federation-wide coordinated parameter selection with client-specific adaptive partitioning. This joint design avoids the inefficiencies of static CKKS configurations while preventing the ring incompatibility issues that arise from heterogeneous parameter choices. Ultimately, DAPHE provides an efficient encrypted communication mechanism that remains fully compatible with HMAGT and supports the consistency evaluation required in FRAHIP.

### Hierarchical multi-scale adaptive graph transformer

Traditional GNN architectures present a structural bottleneck for secure deployment. Although federated learning allows local plaintext training, deep GNNs typically rely on recursive neighborhood aggregation. Despite being efficient for plaintext computation, this recursive structure creates a bottleneck for secure model deployment, where network depth linearly increases the multiplicative depth of homomorphic circuits. For instance, if we strictly execute a 3-layer GCN over encrypted data, the accumulated noise by Layer 3 often exceeds CKKS decryption bounds, requiring expensive bootstrapping or rendering results unusable.

To address this limitation, HMAGT employs a hybrid three-stage architecture that combines encryption-aware graph aggregation with global relationship modeling: (1) Parallel Multi-scale Aggregation for extraction of local structural patterns with constant multiplicative depth; (2) Hierarchical Pooling for informative node selection; and (3) Transformer-based Global Refinement for capturing long-range semantic dependencies. This design justifies the “Graph Transformer” nomenclature by integrating Transformer-based attention with efficient local graph processing.

**Fig 3:**
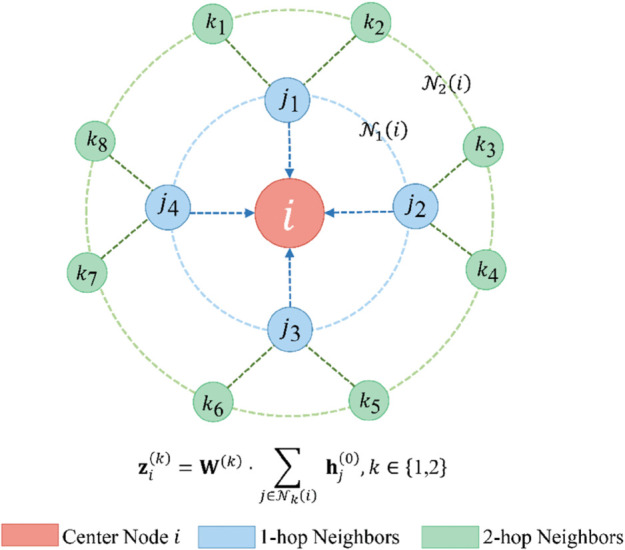
Parallel multi-scale aggregation mechanism in HMAGT. The center node *i* aggregates features from 1-hop neighbors (blue) and 2-hop neighbors (green) in parallel using only the original features ${𝐡}^{\left(0\right)}$, avoiding the depth accumulation found in sequential GNN layers.

**Parallel multi-scale aggregation.** The core innovation lies in the parallel extraction of multi-hop neighborhood information directly from original node features, fundamentally eliminating the sequential depth accumulation inherent in conventional GNN layers. As illustrated in Fig 3, for a center node *i*, the 1-hop neighbor set ${𝒩}_{1}(i)$ and 2-hop neighbor set ${𝒩}_{2}(i)$ contribute features through independent aggregation paths:

$${𝐳}_{i}^{\left(k\right)}={𝐖}^{\left(k\right)}\cdot \sum_{j\in {𝒩}_{k}(i)}{𝐡}_{j}^{\left(0\right)},\;k\in \{1,2\}$$ (4)

where ${𝐡}_{j}^{\left(0\right)}$ denotes the original node feature vector. Critically, because both ${𝐳}_{i}^{\left(1\right)}$ and ${𝐳}_{i}^{\left(2\right)}$ are derived from the same source ${𝐡}^{\left(0\right)}$ via independent linear transformations, the homomorphic multiplicative depth remains constant at one, regardless of the neighborhood radius *k*. This parallel formulation captures multi-scale structural patterns—local connectivity through 1-hop and broader context through 2-hop aggregation—while eliminating the sequential ${𝐡}^{\left(1\right)}\to {𝐡}^{\left(2\right)}$ dependency that would otherwise magnify ciphertext noise. Furthermore, we employ additive aggregation rather than mean aggregation to avoid the additional multiplicative depth and noise accumulation associated with normalization scaling.

Since multi-scale features capture complementary neighborhood patterns, they are fused via linear projection while maintaining dimensional compatibility with DAPHE’s adaptive ring selection:

$${𝐳}_{i}^{\text{fused}}={𝐖}_{\text{fusion}}\cdot \left({𝐳}_{i}^{\left(1\right)}\|{𝐳}_{i}^{\left(2\right)}\right)$$ (5)

where ${𝐖}_{\text{fusion}}\in {\mathbb{R}}^{d\times 2d}$ projects concatenated multi-scale features back to dimension *d*.

**Hierarchical pooling and transformer-based refinement.** While parallel aggregation efficiently captures local structural patterns within a fixed *k*-hop radius, it inherently cannot model semantic dependencies between topologically distant nodes. To address this limitation while managing computational complexity, HMAGT incorporates optimization strategies akin to pruning. Specifically, hierarchical pooling scores nodes based on fused representations, selecting only the top-*K* most informative nodes (${𝒱}_{\text{pool}}$). This effectively prunes redundant or less relevant nodes, reducing the computational complexity of the subsequent attention mechanism from $O(|𝒱{|}^{2})$ to *O*(*K*^2^).

On the pooled node set, HMAGT applies a Transformer encoder layer to enable adaptive cross-node interaction. Let $𝐙\in {\mathbb{R}}^{K\times d}$ denote the stacked representations of selected nodes. The refinement proceeds as:

$${𝐙}^{\prime }=\text{LayerNorm}\left(𝐙+\text{MultiHead}(𝐙,𝐙,𝐙)\right)$$ (6)

$${𝐙}^{\prime \prime }=\text{LayerNorm}\left({𝐙}^{\prime }+\text{FFN}({𝐙}^{\prime })\right)$$ (7)

where MultiHead(𝐐,𝐊,𝐕) computes multi-head self-attention via $\text{softmax}(\frac{𝐐{𝐊}^{T}}{\sqrt{d}})𝐕$, and FFN(·) is a two-layer feed-forward network with residual connections. This architecture allows each node to attend to all other selected nodes, capturing global contextual information that local aggregation alone cannot provide. Critically, all nonlinear operations—including softmax in attention computation and LayerNorm—are executed locally in plaintext at clients before encryption, introducing zero additional homomorphic computation burden.

**Graph-level representation.** For graph classification tasks, the refined node representations are aggregated into a fixed-dimensional graph embedding through homomorphic-efficient global sum pooling:

$${𝐳}_{\text{graph}}=\sum_{i\in {𝒱}_{\text{pool}}}{{𝐳}_{i}^{\prime }}^{\prime },\;𝐲=\text{softmax}({𝐖}_{\text{cls}}{𝐳}_{\text{graph}}+{𝐛}_{\text{cls}})$$ (8)

where global summation introduces no additional noise under homomorphic encryption. Similar to the attention layer, the final classification projection and softmax computation occur locally at clients, ensuring that only gradients are encrypted and transmitted.

Through this end-to-end pipeline—parallel multi-scale aggregation, hierarchical pooling, and Transformer refinement—HMAGT achieves expressive graph representation learning while maintaining a homomorphic multiplicative depth of exactly one (the single matrix multiplication in Eq 4). This design makes privacy-preserving federated GNN training practically feasible without requiring expensive bootstrapping operations.

### Federated robust aggregation via homomorphic inner product

Byzantine-robust aggregation relies on the ability to evaluate the behavioral consistency of participating clients, a task traditionally performed by analyzing gradient similarity in the plaintext domain. Methods such as Krum [27] and geometric median aggregation [28] require access to unencrypted model updates to compute pairwise distances or centralized deviation measures, thereby exposing gradients to reconstruction attacks [32]. Cryptography-oriented approaches mitigate this risk but introduce their own limitations: ELSA [33] depends on an honest-majority assumption across non-colluding servers, whereas RFLPA [34] incorporates reputation information but reveals partial gradient statistics during verification. These designs do not support fully encrypted Byzantine detection, and thus cannot provide privacy-preserving robustness in heterogeneous federated environments.

FRAHIP overcomes these limitations through an encrypted-domain mechanism that reduces high-dimensional similarity computation to scalar-level consistency assessment while preserving the confidentiality of all model updates. The framework begins by computing an encrypted consensus gradient,

$$[{\bar{g}}^{\left(t\right)}]=\frac{1}{K}\sum_{k=1}^{K}[g_{k}^{\left(t\right)}],$$ (9)

and evaluates each participant’s contribution using a homomorphic inner product,

$$[s_{i}]=\langle [g_{i}^{\left(t\right)}],[{\bar{g}}^{\left(t\right)}]\rangle_{\mathrm{HE}}.$$ (10)

This process compresses each *d*-dimensional gradient into a single encrypted scalar representing directional consistency. The server then transmits only the K+1 encrypted values $\left\{[s_{0}],[s_{1}],\ldots ,[s_{K}]\right\}$ to the TPA for decryption, where *[s*_0_*]* denotes the encrypted consensus norm. Since no gradient ciphertexts are ever exposed, information leakage is reduced by several orders of magnitude compared to plaintext similarity evaluation.

Upon receiving the decrypted scalar values, the server computes normalized consistency scores,

$$FS_{i}=\frac{s_{i}}{\sqrt{\|{𝐠}_{i}^{\left(t\right)}{\|}_{2}^{2}\cdot s_{0}}},$$ (11)

which quantify the alignment of each client’s update with the aggregated optimization direction. These instantaneous scores are incorporated into a temporal reputation mechanism that differentiates persistent adversarial behavior from benign stochastic variations:

$${rep}_{i}^{\left(t\right)}=\left\{ \begin{array}{cc} \min({rep}_{i}^{\left(t-1\right)}+\gamma ,{rep}_{\max}) & \text{if }{FS}_{i}\geq \theta ,\\ \max({rep}_{i}^{\left(t-1\right)}-\gamma ,{rep}_{\min}) & \text{if }{FS}_{i}<\theta -\delta ,\\ {rep}_{i}^{\left(t-1\right)} & \text{otherwise}. \end{array}\right.$$ (12)

**Fig 4:**
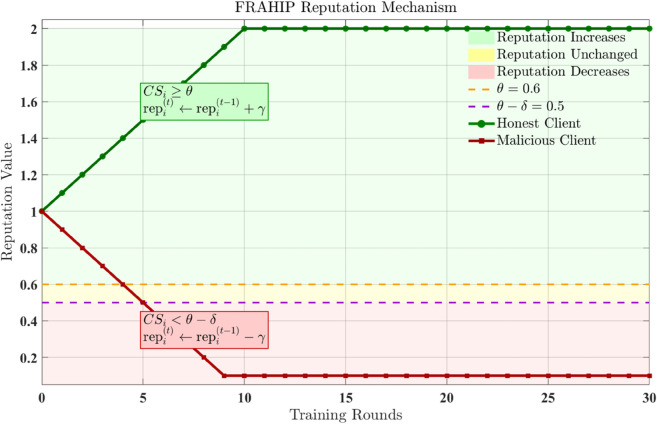
FRAHIP reputation mechanism dynamics. Clients with consistently aligned gradients accumulate reputation, while adversarial or inconsistent behavior results in monotonic decay.

As illustrated in Fig 4, as reputation evolves over rounds, honest participants naturally gain increasing influence, whereas adversarial clients become marginalized. Final aggregation is performed entirely within the encrypted domain using reputation-weighted averaging:

$$[g_{\mathrm{global}}^{\left(t\right)}]=\sum_{i=1}^{K}\frac{\exp(\beta \;{\mathrm{rep}}_{i}^{\left(t\right)})}{\sum_{j=1}^{K}\exp(\beta \;{\mathrm{rep}}_{j}^{\left(t\right)})}\cdot [g_{i}^{\left(t\right)}],$$ (13)

where β controls the sharpness of the weighting distribution. This mechanism guarantees that adversarial influence decreases exponentially over training rounds.

The complete protocol integrating encrypted similarity evaluation, scalar communication, reputation update, and encrypted aggregation is summarized in Algorithm 3. The design ensures that the entire robustness pipeline operates without accessing plaintext gradients, while the TPA observes only 𝒪(K) scalar values per round.

**Algorithm 3 FRAHIP: Federated robust aggregation via homomorphic inner product.**

**Require:** Client set $𝒞=\{C_{1},\ldots ,C_{K}\}$, rounds *T*, parameters γ,θ,δ,β

**Ensure:** Global model $\omega^{\left(T\right)}$

1: Initialize: $rep_{i}^{\left(0\right)}=1.0,\forall i\in [K]$

2: **for** *t* = 1 to *T* **do**

3:   **Phase 1: Local Training and Encryption**

4:   **for** each client *C*_*i*_ **do**

5:    ${𝐠}_{i}^{\left(t\right)}\leftarrow \nabla {ℒ}_{C_{i}}(\omega^{\left(t-1\right)})$

6:    $\left[{𝐠}_{i}^{\left(t\right)}]\leftarrow \text{DAPHE.Encrypt}({𝐠}_{i}^{\left(t\right)}\right)$

7:    Send $\left[{𝐠}_{i}^{\left(t\right)}\right]$ and $\|{𝐠}_{i}^{\left(t\right)}{\|}_{2}^{2}$ to CS

8:   **end for**

9:   **Phase 2: Homomorphic Consensus and Similarity**

10:   $\left[{\bar{𝐠}}^{\left(t\right)}]\leftarrow \frac{1}{K}\sum_{i=1}^{K}[{𝐠}_{i}^{\left(t\right)}\right]$

11:   $[s_{i}]\leftarrow \langle [{𝐠}_{i}^{\left(t\right)}],[{\bar{𝐠}}^{\left(t\right)}]\rangle_{HE}$

12:   **Phase 3: Reputation Update and Aggregation**

13:   TPA decrypts $\left\{[s_{0}],[s_{1}],\ldots ,[s_{K}]\right\}$ and returns scalars

14:   Update $rep_{i}^{\left(t\right)}$ based on consistency scores

15:   $\left[{𝐠}_{global}^{\left(t\right)}]\leftarrow \sum_{i=1}^{K}w_{i}^{\left(t\right)}\cdot [{𝐠}_{i}^{\left(t\right)}\right]$

16:   $\omega^{\left(t\right)}\leftarrow \omega^{\left(t-1\right)}-\eta \cdot {𝐠}_{global}^{\left(t\right)}$

17: **end for**

18: **return** $\omega^{\left(T\right)}$

FRAHIP achieves Byzantine resilience under *f*<*K*/3 adversarial clients while preserving full gradient privacy, with malicious influence decaying as $\sum_{i\in ℳ}w_{i}^{\left(T\right)}\leq |ℳ|\cdot \exp(-\gamma \beta T)$. Experimental results in the Experimental Results section verify that FRAHIP maintains high prediction accuracy despite the presence of Byzantine participants.

### Security analysis

The security analysis of FedGraphHE establishes privacy and robustness guarantees against the defined threat model. The security analysis first defines key parameters used in the evaluation: consistency threshold θ, reputation adjustment step size γ, and temperature parameter β for softmax weighting in the aggregation process.

**Theorem 1** (DAPHE Semantic Security). *Under Ring-LWE hardness assumption, DAPHE maintains IND-CPA security against polynomial-time adversaries with semi-honest servers and up to f<K/3 malicious clients.*

*Proof Sketch:* Security follows from: (1) Dynamic parameter selection operates deterministically on gradient dimensions without accessing values; (2) CKKS encryption with independent keys maintains IND-CPA security; (3) Adaptive partitioning preserves semantic security. ◻

**Theorem 2** (HMAGT Privacy Preservation). *HMAGT preserves semantic security of DAPHE-encrypted inputs against semi-honest adversaries.*

*Proof Sketch:* All homomorphic operations (linear aggregation and multiplication) maintain IND-CPA security guarantees while keeping noise within decryption bounds through HMAGT’s controlled multiplication depth design. ◻

**Theorem 3** (FRAHIP Byzantine robustness). *Under f<K/3 malicious clients, FRAHIP ensures exponential decay of malicious influence $\sum_{i\in ℳ}w_{i}^{\left(T\right)}\leq |ℳ|\cdot \exp(-\gamma \beta T)$ and gradient privacy through 𝒪(K) scalar exposure to TPA.*

*Proof Sketch:* Honest clients achieve higher consistency scores ${\text{FS}}_{i}\geq \theta$ than malicious clients, enabling reputation-based weight decay. TPA access to 𝒪(K) scalars prevents gradient reconstruction from underdetermined systems with d≫K. ◻

**Theorem 4** (End-to-End Security). *FedGraphHE achieves computational security under the ideal-real paradigm against semi-honest servers, semi-honest TPA, and malicious clients.*

*Proof Sketch:* Security under composition follows from DAPHE’s IND-CPA encryption, HMAGT’s privacy preservation, and FRAHIP’s bounded information exposure, with privacy loss limited to inherent federated learning leakage. ◻

The framework provides computational privacy under Ring-LWE assumptions, privacy protection against TPA with 𝒪(K) scalar access, and Byzantine robustness against *f*<*K*/3 malicious clients. For 128-bit security, the framework requires ring dimension N≥16384 and appropriate reputation parameters (θ,γ,β) ensuring convergence. Smaller dimensions (e.g., *N* = 8192) may be acceptable for applications with reduced security requirements.

## 4 Experimental results

### Experimental setup

The experimental setup is summarized in Table 2. The evaluation employs a 70/15/15 stratified split for the ISIC 2020 dataset to preserve balanced class distribution. For citation networks (Cora, CiteSeer, PubMed), standard splits are adapted to our federated setting while maintaining proportional data distribution across participating clients. Detailed parameter settings are provided in Table 3.

**Table 2:** Experimental setup configuration.

Component	Specification
Operating System	Ubuntu 20.04 LTS
Processor	Intel Core i5-12600K
GPU	NVIDIA RTX 3090
Python Version	3.8
Homomorphic Encryption	Microsoft SEAL 3.7.2
Deep Learning Framework	PyTorch 1.9.0
GNNs	PyTorch Geometric 2.0.1

**Table 3:** Core parameters settings.

Parameter	Value
Local epochs *E*	5
Global rounds *T*	30
Data distribution	Non-IID (α=0.5)
Learning rate	0.001
Hidden dimension	128
Security level λ	128-bit
Ring dimension *N*	Dynamic (8192-32768)
Multi-scale kernels	k=[1,2]
Reputation step γ	0.1
Consistency threshold θ	0.6
Threshold tolerance δ	0.1
Temperature parameter β	2.0

The method is evaluated on four real-world datasets, as presented in Table 4. For the ISIC 2020 dataset, dermoscopic images are segmented into patches using a sliding window approach, with node features extracted through pre-trained encoders. For the citation networks (Cora, CiteSeer, and PubMed), TF-IDF vectorization of paper abstracts serves as node features.

**Table 4:** Dataset description.

Dataset	Node	Edge	Feature	Class
ISIC 2020	33126	264968	2048	2
Cora	2708	10556	1433	7
PubMed	19717	44338	500	3
Citeseer	3327	9228	3073	6

### Accuracy evaluation

The effectiveness of FedGraphHE is evaluated across two complementary experimental settings: medical image classification to assess applicability in privacy-sensitive healthcare scenarios, and standard graph node classification benchmarks to evaluate generalization across heterogeneous graph structures. All experiments employ a federated setup with 10 clients over extended training periods. Performance metrics are reported as mean ± standard deviation across five independent runs to ensure statistical reliability.

#### Medical image classification.

FedGraphHE is first evaluated on the ISIC 2020 skin cancer dataset, simulating collaborative training among ten medical institutions under strict privacy constraints. The optimization process exhibits stable convergence, with test accuracy reaching 98.18% and remaining stable thereafter. Table 5 summarizes the classification performance in multiple evaluation metrics, compared with representative federated GNN methods that preserve privacy. A comprehensive comparison across six representative baselines and multiple datasets is provided in Table 6.

**Table 5:** Classification performance on the ISIC 2020 dataset (10 clients, 5 runs).

Method	ACC (%)	Precision (%)	Recall (%)	F1 (%)	AUC (%)
SecGNN [12]	97.62±0.18	97.68±0.45	97.42±0.52	97.55±0.48	98.21±0.35
CryptoGCN [13]	97.78±0.16	97.82±0.41	97.58±0.46	97.70±0.43	98.34±0.31
FedSHE [24]	97.95±0.14	98.02±0.35	97.88±0.39	97.95±0.37	98.52±0.28
FedGraphHE (ours)	98.18±0.16	98.21±0.30	98.09±0.34	98.15±0.32	98.73±0.24

**Table 6:** Accuracy comparison across all datasets (10 clients, 5 runs).

Method	ISIC 2020 (%)	Cora (%)	CiteSeer (%)	PubMed (%)
SecGNN [12]	97.62±0.18	78.5±0.3	77.8±0.4	79.2±0.3
CryptoGCN [13]	97.78±0.16	79.2±0.3	78.5±0.3	79.8±0.3
FedSHE [24]	97.95±0.14	80.8±0.2	80.0±0.3	80.5±0.2
FedPerGNN [15]	97.85±0.15	79.9±0.3	79.2±0.3	79.9±0.3
ESA-FedGNN [16]	97.92±0.14	79.6±0.3	78.8±0.4	79.5±0.3
RFLPA [34]	97.95±0.13	80.2±0.3	79.5±0.3	80.0±0.3
**FedGraphHE (ours)**	98.18±0.16	82.5±0.3	81.3±0.3	81.2±0.3

FedGraphHE achieves the highest performance across all metrics. In particular, the high recall (98.09%) is valuable for clinical applicability, as it reduces the risk of missing malignant lesions. The balanced precision and recall further indicate that FedGraphHE maintains robust classification performance without bias toward either class.

#### Comparative performance analysis.

To comprehensively address baseline coverage and incorporate the latest advances in federated learning, we evaluated FedGraphHE against six representative privacy-preserving federated GNN frameworks spanning from 2022 to 2024. These baselines covered diverse privacy mechanisms and aggregation strategies, ensuring a thorough comparison with current research frontiers: SecGNN [12] and CryptoGCN [13] relied on secure multi-party computation and cryptographic protocols for gradient protection; FedSHE [24] used static homomorphic encryption; FedPerGNN [15] leveraged public subgraph sharing; ESA-FedGNN [16] included adaptive edge sampling; and RFLPA [34], a state-of-the-art framework from NeurIPS 2024, integrated reputation-based client filtering for robustness.

Table 6 presents a comprehensive accuracy comparison across all four datasets. FedGraphHE consistently achieves the highest accuracy on every dataset: 98.18% on ISIC 2020 (medical imaging), 82.5% on Cora, 81.3% on CiteSeer, and 81.2% on PubMed (citation networks). The improvements over the second-best method (FedSHE) are particularly pronounced on Cora and CiteSeer (1.3%–1.7%), where the hierarchical multi-scale aggregation in HMAGT effectively captures multi-hop structural information even under encryption constraints.

The consistent superiority across diverse datasets demonstrates FedGraphHE’s strong generalization capability. Compared with cryptographic baselines (SecGNN, CryptoGCN), FedGraphHE benefits from graph-aware aggregation that better preserves structural information under encryption. Compared with the static encryption baseline (FedSHE), FedGraphHE achieves higher accuracy while maintaining competitive efficiency through dynamic ciphertext parameter selection in DAPHE.

#### Node classification performance.

**Fig 5:**
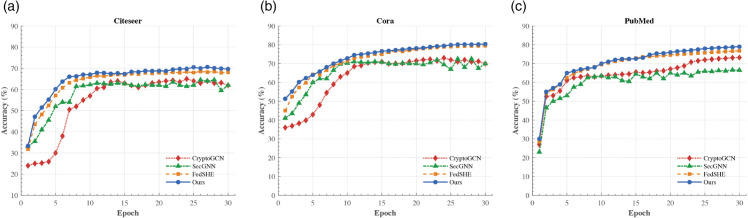
Node classification accuracy across three benchmark datasets over training epochs. FedGraphHE achieves faster convergence and higher accuracy compared to baselines.

FedGraphHE demonstrates competitive performance on standard graph learning benchmarks (Cora, CiteSeer, and PubMed) compared to existing privacy-preserving methods. As illustrated in Fig 5, FedGraphHE achieves faster convergence and higher final accuracy, with particularly notable improvements in the early training phases across all citation network datasets.

### Ablation study

To systematically assess the contribution of each component, we conduct ablation experiments across four datasets under both benign and adversarial settings. We evaluate five variants: the full FedGraphHE framework and four ablated versions removing (i) DAPHE (using a fixed ring dimension *N* = 16384), (ii) HMAGT (using a standard 2-layer GCN), (iii) FRAHIP (replacing the aggregation with simple FedAvg), or (iv) only the reputation mechanism (retaining homomorphic inner-product aggregation but disabling adaptive client weighting). Table 7 reports clean accuracy for all datasets and robustness metrics under Byzantine attacks on ISIC 2020 with *f* = 3 malicious clients.

**Table 7:** Ablation study results across four datasets (10 clients, 5 runs).

Variant	Clean Accuracy (%)				ISIC Attack (*f* *= 3)*	
	ISIC	Cora	CiteSeer	PubMed	ACC (%)	ASR (%)
Full	98.18±0.16	82.5±0.3	81.3±0.3	81.2±0.3	95.82	3.15
without DAPHE	$97.78\pm 0.17^{*}$	$81.8\pm 0.3^{*}$	$80.7\pm 0.3^{*}$	$80.6\pm 0.3^{*}$	95.35	3.62
without HMAGT	$96.08\pm 0.22^{**}$	$79.2\pm 0.4^{**}$	$78.0\pm 0.4^{**}$	$78.2\pm 0.4^{**}$	92.85	5.28
without FRAHIP	$97.92\pm 0.16^{ns}$	$82.0\pm 0.3^{ns}$	$80.9\pm 0.3^{ns}$	$80.8\pm 0.3^{ns}$	87.08	11.28
without Reputation	$97.42\pm 0.18^{*}$	$81.4\pm 0.3^{*}$	$80.4\pm 0.3^{*}$	$80.2\pm 0.3^{*}$	90.85	7.12

*p < 0.05, **p < 0.01, ^ns^not significant (paired t-test vs. Full). ASR: Attack Success Rate under model poisoning (λ=1.0).

The ablation results demonstrate the distinct role of each module. Removing HMAGT causes the largest drop in clean accuracy (*p* < 0.01), which confirms that the multi-scale aggregation is essential for learning complex structural features, especially in citation networks. This shows that standard GCN layers are insufficient for capturing multi-hop dependencies under the constraints of encrypted training.

Regarding robustness, disabling FRAHIP results in a sharp increase in attack success rate (up to 11.28%), validating it as the primary defense against Byzantine attacks. It is worth noting that the “without Reputation” variant shows a slight decrease in clean accuracy compared to the full model. This suggests that without the smoothing effect of historical reputation, the raw consistency scores can fluctuate, occasionally penalizing benign updates. The reputation mechanism therefore acts as a stabilizer, ensuring that only truly malicious gradients are suppressed while preserving normal training dynamics.

Finally, removing DAPHE leads to a statistically significant drop in accuracy (*p* < 0.05). This indicates that the benefit of DAPHE extends beyond communication efficiency. By adapting the ring dimension to the data, it avoids the quantization noise often caused by suboptimal fixed parameters, thereby maintaining higher gradient precision across heterogeneous clients.

### Robustness evaluation

We evaluate the Byzantine robustness of FRAHIP under the standard constraint *f*<*K*/3 with *K* = 10 clients, allowing up to *f* = 3 malicious participants. To ensure comprehensive comparison spanning both classical and state-of-the-art approaches, FRAHIP is compared against three representative robust aggregation methods: Krum [27] (NIPS 2017, a classical distance-based method), FLTrust [35] (NDSS 2021, a trust-score approach), and RFLPA [34] (NeurIPS 2024, the current state-of-the-art incorporating reputation mechanisms similar to our approach). The evaluation is conducted on the ISIC 2020 dataset using a federated configuration with enhanced adversarial challenges.

**Attack configuration.** We consider two Byzantine attack types: (i) model poisoning via gradient sign flipping ${𝐠}_{i}^{\left(t\right)}\leftarrow -\lambda {𝐠}_{i}^{\left(t\right)}$ with λ=1.0, and (ii) data poisoning via label manipulation. The attack success rate (ASR) is defined as:

$$\text{ASR}=\frac{{\text{ACC}}_{\text{clean}}-{\text{ACC}}_{\text{attack}}}{{\text{ACC}}_{\text{clean}}}\times 100\%.$$ (14)

As reported in Table 8, FRAHIP maintains accuracy above 95% with ASR below 2.5% under the strongest threat (*f* = 3), consistently outperforming Krum, FLTrust, and RFLPA across all Byzantine settings. The robustness gains originate from encrypted gradient consistency evaluation through homomorphic inner products $\left[\langle {𝐠}_{i}^{\left(t\right)},{\overset{―}{𝐠}}^{\left(t\right)}\rangle \right]$ and adaptive reputation weighting, which jointly suppress malicious updates and stabilize aggregation over time.

**Table 8:** Robustness under Byzantine attacks (ISIC 2020, 10 clients).

Byzantine		FRAHIP		RFLPA		Krum		FLTrust	
*f*	Type	ACC (%)	ASR (%)	ACC (%)	ASR (%)	ACC (%)	ASR (%)	ACC (%)	ASR (%)
0	Baseline	98.18	0	98.18	0	98.18	0	98.18	0
1	Model Poisoning	97.4±0.2	0.8	96.2±0.3	1.9	94.2±0.8	4.1	95.9±0.5	2.3
	Data Poisoning	97.9±0.1	0.3	96.8±0.2	1.3	93.7±0.9	4.7	94.9±0.6	3.4
2	Model Poisoning	96.8±0.2	1.4	94.8±0.4	3.4	91.5±1.1	6.8	93.6±0.7	4.7
	Data Poisoning	97.2±0.2	1.0	95.2±0.3	3.0	90.9±1.2	7.5	92.9±0.8	5.3
3	Model Poisoning	95.9±0.2	2.3	93.8±0.5	4.4	89.7±1.4	8.6	92.4±1.0	5.9
	Data Poisoning	96.3±0.2	1.9	94.2±0.4	4.0	88.4±1.5	10.0	91.3±1.0	7.0

### Efficiency and communication analysis

Communication overhead in federated learning primarily arises from encrypted parameter transmission between clients and the central server. The optimization effectiveness of the DAPHE module is evaluated by comparing FedGraphHE with FedAvg (the non-encrypted baseline) and FedSHE (the homomorphic encryption baseline) across multiple datasets.

The DAPHE module addresses a fundamental challenge in homomorphic encryption schemes: static ring dimension selection often leads to suboptimal slot utilization when client gradients have heterogeneous dimensions. By dynamically adapting ring dimensions $N_{l}=N(d_{l})$ based on federation-wide gradient characteristics, DAPHE significantly reduces communication volume while maintaining cryptographic security [23].

**Fig 6:**
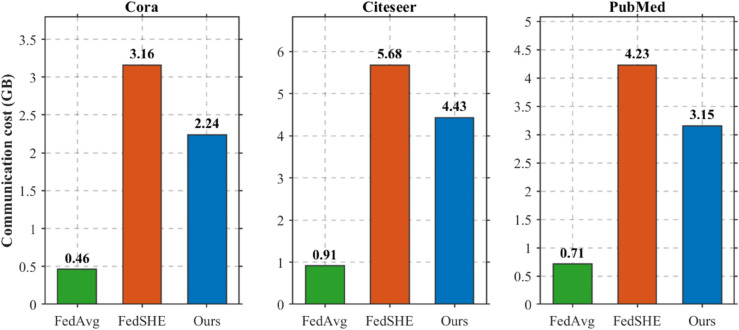
Communication cost comparison.

Fig 6 demonstrates the communication cost comparison results. FedGraphHE consistently achieves substantial reductions in communication volume compared to FedSHE across all evaluated datasets, with improvements ranging from 20–30%. This reduction primarily stems from improved slot utilization efficiency and adaptive partitioning strategies that minimize padding overhead.

Tables 9 and 10 present the temporal analysis of training efficiency. The results indicate that FedGraphHE’s efficiency advantage becomes more pronounced as training progresses, with communication time reductions typically exceeding 50% compared to FedSHE in later rounds. The training time analysis demonstrates similar trends, confirming the practical benefits of the DAPHE optimization.

**Table 9:** Communication time (seconds).

Dataset & Method		Epoch								
		1	3	6	9	12	15	18	21	24
ISIC 2020	FedAvg	0.15	0.42	0.89	1.34	1.81	2.25	2.72	3.18	3.64
	FedSHE	18.7	58.3	115.2	174.8	233.1	292.5	351.8	410.6	469.9
	FedGraphHE	9.8	30.6	60.4	91.2	121.3	152.1	183.7	214.2	244.7
Cora	FedAvg	0.09	0.26	0.54	0.81	1.09	1.36	1.64	1.91	2.18
	FedSHE	9.8	30.6	60.4	91.2	121.8	152.6	183.2	214.1	244.7
	FedGraphHE	4.5	14.2	28.1	42.6	56.8	71.3	85.6	100.2	114.7
Citeseer	FedAvg	0.11	0.32	0.67	1.01	1.36	1.7	2.05	2.39	2.74
	FedSHE	12.3	38.4	75.8	114.6	153.2	192.1	230.8	269.9	308.7
	FedGraphHE	6.1	19.2	38	57.4	76.6	96.1	115.4	135	154.5
PubMed	FedAvg	0.16	0.46	0.96	1.45	1.95	2.44	2.94	3.43	3.93
	FedSHE	15.6	48.7	96.2	145.3	194.1	243.4	292.5	341.9	391.2
	FedGraphHE	7.8	24.5	48.4	73.1	97.6	122.4	147.3	171.9	196.7

**Table 10:** Training Time (seconds).

Dataset & Method		Epoch								
		1	3	6	9	12	15	18	21	24
ISIC 2020	FedAvg	2.3	6.4	13.1	19.5	26.2	32.6	39.3	45.7	52.4
	FedSHE	156.3	472.6	954.8	1438.2	1920.5	2403.8	2886.1	3369.4	3851.7
	FedGraphHE	67.2	201.8	405.3	609.7	814.1	1018.6	1223	1427.4	1631.8
Cora	FedAvg	1.4	4.1	8.4	12.6	16.9	21.1	25.4	29.6	33.9
	FedSHE	78.4	236.9	479.2	722.6	965.9	1209.2	1452.5	1695.8	1939.1
	FedGraphHE	36.8	111.2	224.7	339.1	453.5	567.9	682.3	796.7	911.1
Citeseer	FedAvg	1.8	5.2	10.7	16.1	21.6	27	32.5	37.9	43.4
	FedSHE	98.7	298.4	603.2	909.1	1214.9	1520.7	1826.5	2132.3	2438.1
	FedGraphHE	47.1	142.4	287.9	434.5	581	727.5	874	1020.5	1167
PubMed	FedAvg	2.1	6	12.3	18.5	24.8	31	37.3	43.5	49.8
	FedSHE	126.9	383.8	775.4	1168.1	1560.7	1953.3	2345.9	2738.5	3131.1
	FedGraphHE	58.6	177.4	358.3	540.2	722.1	904.6	1085.9	1267.8	1449.7

While homomorphic encryption inevitably introduces computational overhead compared to plaintext transmission, FedGraphHE demonstrates that careful parameter optimization can significantly mitigate these costs. The experimental validation confirms that DAPHE effectively balances privacy preservation with practical efficiency, making privacy-preserving federated graph learning more viable for real-world deployment.

## 5 Conclusion and future work

This paper presents FedGraphHE, a privacy-preserving federated graph neural network framework that addresses the critical challenges facing collaborative intelligence in distributed environments, particularly in smart healthcare networks where medical institutions require secure joint learning from distributed patient data. The framework integrates three synergistic modules: DAPHE provides dynamic encryption optimization to reduce computational overhead from homomorphic encryption, HMAGT enables encryption-aware graph processing to address gradient privacy vulnerabilities, and FRAHIP delivers Byzantine-resilient aggregation to counter malicious participants, maintaining over 95% accuracy under *f*<*K*/3 Byzantine attacks.

Experimental results demonstrate FedGraphHE’s effectiveness across diverse application scenarios. On medical image classification tasks (ISIC 2020), the framework achieves 98.18% accuracy, while on citation network datasets, it delivers accuracy equivalent to centralized methods and significantly outperforms existing privacy-preserving approaches. However, several limitations warrant acknowledgment. The framework incurs a 27–31× computational overhead compared to plaintext methods, which can restrict the deployment on devices limited by resources. Additionally, the dependence on a TPA for scalar decryption makes the framework incompatible with fully decentralized scenarios. The current design is also specific to static graph structures, requiring adaptation for dynamic topologies.

Future research directions should address these limitations through multiple avenues. Investigating threshold cryptography could eliminate TPA dependencies, while developing lightweight implementations would enable edge deployment. Extending the framework support to dynamic graph learning scenarios would broaden its applicability and enhance its practical utility for secure collaborative learning in privacy-sensitive applications.
